# Phenotypic and Functional Characterization of Mesenchymal Stem/Multipotent Stromal Cells from *Decidua Basalis* of Human Term Placenta

**DOI:** 10.1155/2016/5184601

**Published:** 2016-02-10

**Authors:** F. M. Abomaray, M. A. Al Jumah, K. O. Alsaad, D. Jawdat, A. Al Khaldi, A. S. AlAskar, S. Al Harthy, A. M. Al Subayyil, T. Khatlani, A. O. Alawad, A. Alkushi, B. Kalionis, M. H. Abumaree

**Affiliations:** ^1^King Abdullah International Medical Research Center, Mail Code 1515, P.O. Box 22490, Riyadh 11426, Saudi Arabia; ^2^Department of Clinical Science, Intervention and Technology, Division of Obstetrics and Gynecology, Karolinska Institutet, 14186 Stockholm, Sweden; ^3^Center for Hematology and Regenerative Medicine, Karolinska Institutet, 14186 Stockholm, Sweden; ^4^King Abdulaziz Medical City, Department of Pathology, P.O. Box 22490, Riyadh 11426, Saudi Arabia; ^5^King Abdulaziz Medical City, Division of Cardiac Surgery, P.O. Box 22490, Riyadh, Saudi Arabia; ^6^National Center for Stem Cell Technology, Life Sciences and Environment Research Institute, King Abdulaziz City for Science and Technology, P.O. Box 6086, Riyadh 11442, Saudi Arabia; ^7^College of Science and Health Professions, King Saud bin Abdulaziz University for Health Sciences, Mail Code 3124, P.O. Box 3660, Riyadh 11481, Saudi Arabia; ^8^Department of Obstetrics and Gynaecology, University of Melbourne and Department of Perinatal Medicine Pregnancy Research Centre, Royal Women's Hospital, Parkville, VIC 3052, Australia

## Abstract

Mesenchymal stem cell (MSC) therapies for the treatment of diseases associated with inflammation and oxidative stress employ primarily bone marrow MSCs (BMMSCs) and other MSC types such as MSC from the chorionic villi of human term placentae (pMSCs). These MSCs are not derived from microenvironments associated with inflammation and oxidative stress, unlike MSCs from the* decidua basalis *of the human term placenta (DBMSCs). DBMSCs were isolated and then extensively characterized. Differentiation of DBMSCs into three mesenchymal lineages (adipocytes, osteocytes, and chondrocytes) was performed. Real-time polymerase chain reaction (PCR) and flow cytometry techniques were also used to characterize the gene and protein expression profiles of DBMSCs, respectively. In addition, sandwich enzyme-linked immunosorbent assay (ELISA) was performed to detect proteins secreted by DBMSCs. Finally, the migration and proliferation abilities of DBMSCs were also determined. DBMSCs were positive for MSC markers and HLA-ABC. DBMSCs were negative for hematopoietic and endothelial markers, costimulatory molecules, and HLA-DR. Functionally, DBMSCs differentiated into three mesenchymal lineages, proliferated, and migrated in response to a number of stimuli. Most importantly, these cells express and secrete a distinct combination of cytokines, growth factors, and immune molecules that reflect their unique microenvironment. Therefore, DBMSCs could be attractive, alternative candidates for MSC-based therapies that treat diseases associated with inflammation and oxidative stress.

## 1. Introduction

Mesenchymal stem cells (MSCs) are multipotent adult cells that were originally described as plastic-adherent, fibroblast-like cells derived from bone marrow (BM) [1]. MSCs have extensive self-renewal properties, can form fibroblast-like colonies* in vitro* called colony-forming unit fibroblasts (CFU-F), and have potential to differentiate into mesenchymal lineage cells such as osteocytes, adipocytes, and chondrocytes [1, 2].

Currently, BM-derived MSCs (BMMSCs) are the most widely used stem cell type in clinical trials. However, BMMSCs have significant limitations including the invasive harvesting method used for collection, insufficient numbers of stem cells in adult BM (approximately 0.001–0.01%), and reduction in cell numbers and differentiation potential with increasing age of the donor [3]. These limitations of BMMSCs for cell-based therapies prompted the isolation and characterization of MSCs from many other adult and fetal tissues, such as liver, dental pulp, adipose tissue, endometrium, muscle, amniotic fluid, placenta, and umbilical cord blood [4–10]. The inconsistent methods and marker antibodies used to isolate and characterise MSCs, respectively, prompted The International Society of Cellular Therapy to standardise the minimal criteria to identify MSCs [11]. The term placenta [source of fetal chorionic villi MSC (called pMSCs or CMSCs)] and attached maternal* decidua basalis* [source of* decidua basalis* MSCs (DBMSCs)] are particularly attractive alternate MSC sources because they are readily accessible, abundant, and commonly discarded after normal delivery.

Many MSC-based therapies are directed toward diseases and disorders caused by oxidative stress and associated with increased inflammation, which include atherosclerosis, Alzheimer's disease, Parkinson's disease, neurodevelopmental disorders, angina, thrombosis, and hypertension [12–14]. The rationale for these therapies is that in response to various circulating stimuli including cytokines, chemokines, and growth factors, MSCs migrate to sites of inflammation and injured tissue. At these locations, MSCs must repair the damaged region under conditions of inflammation and oxidative stress, either by engrafting and differentiating into tissue-specific cell types or by paracrine mechanisms where they stimulate endogenous stem cells and/or modulate the functions of immune cells, such as monocytes, macrophages, dendritic cells (DCs), and T and B cells as well as natural killer cells (NK) [15–19].

BMMSCs in their niche are normally exposed to low levels of oxidative stress and only experience increased oxidative stress following injury or disease [20]. Preconditioning BMMSCs and other MSC types by exposure to hypoxic, oxidative stress-inducing conditions improves many important stem cell characteristics [21]. Surprisingly little is known about the properties of MSCs derived from a niche normally exposed to high levels of inflammation and oxidative stress. The expectation is that these MSCs would show significant differences in oxidative stress response as well as cytokines/growth factors/immunomodulatory factors compared to that of BMMSCs which may be equal or more effective than BMMSCs in the therapeutic setting.

In this work we focus on MSCs derived from the* decidua basalis*, which is a maternal tissue exposed to increased levels of inflammation and oxidative stress throughout human pregnancy [22, 23]. The* decidua basalis* comprises a thin layer of maternal endometrial tissue that undergoes structural and functional transformation during early pregnancy. The* decidua basalis* is subsequently invaded by specialized placental trophoblast cells, which adheres the placenta to the* decidua basalis* and underlying myometrium. The* decidua basalis* forms part of the maternal-fetal interface (also referred to as the attachment site of the placenta, or the basal plate), which is composed of maternal* decidua basalis* and fetal villous tissue derived from the chorionic sac. We showed that both maternal* decidua basalis *MSCs (DBMSCs) and fetal chorionic villous MSCs (i.e., pMSCs) were located within vascular microenvironments (i.e., niches) [24, 25]. However, the niches for DBMSCs and pMSCs are very different in their exposure to oxidative stress and inflammatory factors within the maternal and fetal circulations, respectively. The vascular DBMSC niche is directly exposed to maternal blood containing high levels of circulating inflammatory factors and reactive oxygen species during pregnancy. The pMSC niche, in the fetal chorionic villi, on the other hand, is exposed to the fetal circulation, which experiences lower levels of inflammation and oxidative stress [26]. The high resistance of DBMSCs to oxidative stress has been demonstrated in cell culture studies [27]. In a related study, we used the aldehyde dehydrogenase oxidative stress response assay to confirm that DBMSCs in cell culture were more highly resistant to oxidative stress than pMSCs (Kusuma, Abumaree, Pertile, Perkins, Brennecke and Kalionis,* Placenta*, Submitted).

While there is progress in determining the oxidative stress response of DBMSCs, we still lack a comprehensive understanding of their phenotype and growth properties. Moreover, very few studies have addressed the identification of the particular combination of cytokines, growth factors, and immune molecules expressed by term DBMSCs [27, 28]. Such studies have been carried extensively on BMMSCs and other MSC types [29], and we showed that pMSCs express a distinct combination of cytokines, growth factors, and immune molecules and had specific immunomodulatory effects on immune cells including macrophages, dendritic cells, and T cells [16, 18, 19].

Therefore, in this study, we isolated and characterized DBMSCs from the* decidua basalis* that remains attached to the placenta following delivery. The aim of the study was to characterize the phenotypic properties of DBMSCs including their expression of adhesion molecules, chemokines/receptors, cytokines/receptors, and growth factors. In addition, we carried out a functional analysis of DBMSCs where we examined their proliferative response to various cytokines, and their migratory response to chemotactic factors* in vitro*. Our study demonstrated that DBMSCs express a unique combination of molecules involved in many important cellular functions including homing/migration, proliferation, differentiation, immunomodulation, and angiogenesis. These data suggest that MSCs from* decidua basalis* have unique phenotypic and functional properties that make them a potentially important source of MSCs for cell-based therapy.

## 2. Materials and Methods 

### 2.1. Ethics of Experimentation

This study was approved by the institutional research board (Reference # IRBC/246/13) at King Abdulla International Medical Research Centre/King Abdulaziz Medical City, Riyadh, Saudi Arabia. All placentae were obtained with informed consent.

### 2.2. Placentae

Human placentae were obtained from uncomplicated pregnancies following normal vaginal delivery (38–40 weeks of gestation). The gestational age and fetal viability of all pregnancies were confirmed by early ultrasound examination before 20 weeks of gestation. The placentae were used within 2 h of delivery.

### 2.3. *Decidua Basalis*-Derived Mesenchymal Stem Cells (DBMSCs) Isolation and Culture

MSCs were isolated from the* decidua basalis* attached to the maternal side of the human placenta as previously described [7] with the following modifications. Briefly, 10 grams of the decidua tissue was dissected from the maternal surface of placenta and washed thoroughly with phosphate buffered saline (PBS, pH 7.4) to remove excess blood. The tissue was then finely minced and washed with PBS until the fluid was free of blood. After centrifugation at 300 ×g for 5 minutes, the tissue pellet was digested using a digestion solution containing 0.3% collagenase type I (Life Technology, Saudi Arabia) diluted in PBS, 100 *μ*g/mL streptomycin, 100 U/mL Penicillin, and 271 units/mL DNase I (Life Technology, Saudi Arabia) at 37°C in a water bath for 1 hour. The mixture was then filtered with a 100 *μ*m nylon filter (Becton Dickinson, Saudi Arabia), centrifuged and incubated with red blood cell lysing buffer (#sc-3621, FCM Lysing solution, Santa Cruz, Saudi Arabia) for 45 minutes at room temperature (RT) to lyse red blood cells. After centrifugation, the cells were washed and 1 × 10^5^ cells were cultured in T25 flasks (Becton Dickinson, Saudi Arabia) in complete DBMSC culture medium containing Dulbecco's Modified Eagle Medium nutrient mixture F-12 (DMEM-F12), 10% Mesenchymal Stem Cell Certified Fetal Bovine Serum (MSC FBS) (Life Technology, Saudi Arabia), 100 *μ*g/mL L-glutamate, 100 *μ*g/mL streptomycin, and 100 U/mL Penicillin. Cells were then incubated at 37°C in a humidified atmosphere containing 5% CO_2_ and 95% air. When cells reached 75% confluency, they were harvested with TrypLE express detachment solution (Life Technology, Saudi Arabia) and counted using Trypan blue on a haemocytometer chamber. Cells at a density of 1 × 10^5^ cells were recultured in 75 cm^2^ flask (Becton Dickinson, Saudi Arabia) until they reached 75% confluency and were then used in subsequent experiments. Cells were visualized under an inverted Nikon ECLIPSE Ti U microscope (Nikon, Saudi Arabia) and photomicrographs were recorded using Nikon DS-Qi1 camera and Software (Nikon, Saudi Arabia). Twenty placentae were used in this study. Cells from passages 3–5 were used in subsequent experiments.

### 2.4. Flow Cytometry

MSCs expanded in culture from passages 3–5 were phenotypically characterized by flow cytometry as previously published [16]. Briefly, cells were harvested using TrypLE™ Express detachment solution and 1 × 10^5^ of cells were then stained with monoclonal antibodies for 30 minutes. Cells were washed twice by adding cold PBS, pH 7.4, and centrifuged at 150 ×g for 5 minutes at 8°C. To analyse intracellular expression of proteins, cells were fixed with 4% paraformaldehyde in sterile PBS, pH 7.4 for 10 minutes at room temperature and then permeabilized using sterile PBS, pH 7.4 containing 0.1% saponin for 5 minutes at room temperature. Unstained and isotype controls were used. Immunoreactivity to cell surface antibody markers or intracellular proteins was assayed by a BD FACS CANTO II (Becton Dickinson, Saudi Arabia) flow cytometer.

### 2.5. Colony Forming Unit (CFU) Assay

Colony forming efficiency of DBMSCs was assessed as previously published [16]. Briefly, DBMSCs were seeded into six-well plates at a density of 100 cells/well in complete DBMSC culture medium. The medium was then replaced with fresh medium every 3 days. After 14 days culture as described above, the medium was removed and the cells were then washed with PBS for three times. Cells were then fixed with 4% paraformaldehyde in PBS, pH 7.4 for 30 minutes at RT. After washing cells twice with PBS, they were stained with 0.1% crystal violet (Santa Cruz, Saudi Arabia) for 15 minutes at RT, rinsed with distilled water, and visualized and photomicrographs were recorded as described above. Colonies of cell aggregates of ≥50 cells were scored. Each experiment was performed in triplicate using DBMSCs from passages three to five, from twenty individual placentae.

### 2.6. Osteogenic and Adipogenic Induction

Osteogenic and adipogenic differentiation of DBMSCs were assessed as previously published [16]. Briefly, DBMSCs were seeded into six-well plates at a density of 10^4^ cells/well for osteogenic and adipogenic induction in complete DBMSC culture medium and then cultured as described in Section 2.3. When cells reached 70% and 100% confluency for osteogenic and adipogenic induction, respectively, the medium was removed and the cells were washed twice with PBS and then cultured in osteogenic differentiation medium containing osteogenic supplement (Part # 390416, R&D systems, Saudi Arabia) or in adipogenic differentiation medium containing adipogenic supplement (Part # 390415, R&D systems, Saudi Arabia) in complete DBMSC culture medium. Cells cultured without osteogenic or adipogenic differentiation medium were used as controls for osteogenic and adipogenic differentiation. Every three days, the medium was replaced with fresh medium. After 21 days in culture, osteocytes and adipocytes were washed twice with PBS, fixed with 4% paraformaldehyde in PBS, pH 7.4 for 20 minutes at RT, and then washed twice with PBS. For osteocyte staining, cells cultured with or without osteogenic differentiation medium were stained with 2% Alizarin Red S solution, pH 4.2 for 3 minutes at RT, rinsed three times with distilled water. For adipocyte staining, cells cultured with or without adipogenic differentiation medium were incubated with 100-fold dilution LipidTOX*™* Green Neutral Lipid Stain (Life Technology, Saudi Arabia) for 30 minutes in the dark at RT and then were rinsed twice with PBS. Cells were then visualized and recorded as described above. Each experiment was performed in duplicate using DBMSCs from passage three of five individual placentae.

### 2.7. Chondrogenic Induction

Chondrogenic differentiation of DBMSCs was assessed as previously published [16]. Briefly, DBMSCs at density of 2.5 × 10^5^ in a 15 mL conical tube containing complete DBMSC culture medium were centrifuged at 200 ×g for 5 minutes at RT. After removing the culture medium, cells were resuspended in culture medium and centrifuged at 200 ×g for 5 minutes at RT. After removing the medium, cells were resuspended in chondrogenic differentiation medium containing chondrogenic supplement (Part # 390417, R&D systems, Saudi Arabia) in complete DBMSC culture medium. Cells cultured without chondrogenic differentiation medium were used as a control for chondrogenic differentiation. Then, cells were centrifuged at 200 ×g for 5 minutes at RT. After centrifugation, the cap was loosened to allow gas exchange; the cell pellet in the tube was then incubated as described in Section 2.3. Every three days, the medium was replaced with fresh medium without disturbing the cell pellet. After incubation for 21 days, the chondrocyte pellet or the cell pellet from the control was gently washed twice with PBS and frozen in cryoembedding medium (Leica Microsystems, Saudi Arabia) using liquid nitrogen. The frozen chondrocyte blocks or the cell pellet blocks from the control were cut into 10 *μ*m serial sections using a cryostat (Thermo Scientific, Saudi Arabia) and collected on glass slides (Leica Microsystems, Saudi Arabia). After fixing slides in 4% (w/v) paraformaldehyde in PBS, pH 7.4 for 20 minutes at RT, slides were washed twice with PBS and stained with 1% Alcian Blue solution prepared in 0.1 N HCL for 30 minutes at RT. Then, slides were rinsed with 0.1 N HCl for three times and the acidity was neutralized using distilled water. Slides were visualized under the light microscope and images were captured. The number of replicates and passages and numbers of placentae were as indicated in Section 2.6.

### 2.8. Gene Expression Profile

The expression of genes by DBMSCs was determined using QuantiTect Primer Assay (Qiagen, Saudi Arabia) in a real-time polymerase chain reaction (RT-PCR) as previously published [16]. Briefly, total RNA from cells was isolated and cDNA was then synthesized using FastLane Cell cDNA kit and RT Primer Mix (Qiagen, Saudi Arabia) at 42°C for 30 minutes as instructed by the manufacturer. Following quantifying mRNA using QuantiTect SYBR Green PCR Kit (Qiagen, Saudi Arabia) as instructed by the manufacturer, the real-time PCR reaction was carried out in triplicate on the CFX96 real-time PCR detection system (BIO-RAD, Saudi Arabia). Then, the data were analysed using the CFX manager software (BIO-RAD, Saudi Arabia). The gene expression was assessed using the following criteria for CT (cycle threshold) values: CT value > 40, expression considered negative (−); CT between 36 and 40, expression considered weak (+); CT value between 29 and 35, expression considered moderate (++); and CT ≤28, expression considered strong (+++). The relative expression level of the housekeeping gene *β*-actin or GAPDH or 18S rRNA was used as previously described [16]. Each experiment was performed using DBMSCs from passages three to five, using five individual placentae.

### 2.9. Enzyme-Linked Immunosorbent Assay (ELISA)

Secretion of human IL1*β*, IL1Ra, IL-3, IL-5, IL-6, sIL-6R, IL-10, IL12p70, IL-19, vascular endothelial growth factor (VEGF), soluble VEGF Receptor (sVEGFR), interferon gamma (IFN*γ*), macrophage colony-stimulating factor (M-CSF), granulocyte-macrophage colony-stimulating factor (GM-CSF), macrophage migration inhibitory factor (MIF), hepatocyte growth factor (HGF), transforming growth factor beta-1 (TGF*β*-1), leukemia inhibitory factor (LIF), B7H4, indoleamine-pyrrole 2,3-dioxygenase (IDO), and epidermal growth factor (EGF) by DBMSCs was assayed by ELISA kits following the manufacturer's instructions. DBMSC culture medium was prepared as previously described [16]. Briefly, cells were washed three times with PBS and then cultured for 48 hours at a density of 2 × 10^6^ in a 6-well plate containing complete DBMSC culture medium as described in Section 2.3. Conditioned media were then collected by centrifugation at 300 ×g for 10 minutes and analysed by the quantitative sandwich immunoassay. Culture medium not incubated with cells was used as the negative control. ELISA kits were purchased from R&D Systems (Saudi Arabia), Life Technology (Saudi Arabia), and MyBioSource (Saudi Arabia). Each experiment was performed in duplicate using DBMSCs from passages three of five individual placentae.

### 2.10. Cell Proliferation Assay

The effect of selected cytokines on the proliferation of DBMSCs was studied by seeding cells at a density of 2 × 10^3^ per well in 96-well tissue culture plates containing complete DBMSC culture medium. Five human recombinant cytokines [IL-4 (3 ng/mL), HGF (400 ng/mL), IL-6 (10 ng/mL), Rantes (40 ng/mL), and IL7A (40 ng/mL)] were assessed. Cytokines were dissolved in complete DBMSC culture medium and then added to the cell culture and incubated for 48 hours. The concentrations used gave an optimal effect as shown in a dose response experiment (data not shown) and these concentrations were consistent with our published results [16]. Cell proliferation was assessed using a tetrazolium compound [3-(4,5-dimethylthiazol-2-yl)-5-(3-carboxymethoxyphenyl)-2-(4-sulfophenyl)-2H-tetrazolium, inner salt; MTS] kit (#G5421, CellTiter 96® Aqueous Non-Radioactive Cell Proliferation Assay, Promega, Saudi Arabia) according to the manufacturer's instructions. Briefly, MTS solution was added to each well of the 96-well assay plate containing cells in complete DBMSC culture medium in presence or absence of cytokines, incubated for 4 hours. The absorbance at 490 nm was recorded using an ELISA plate reader (Spectra MR, Dynex Technologies, Saudi Arabia). Results are presented as means (± SD) obtained from triplicate cultures. MTS solution in medium not exposed to cells was used as blank. Each experiment was performed in triplicate using DBMSCs from passages three to five, from five individual placentae.

### 2.11. Cell Migration Assay

DBMSC migration in response to selected chemotactic factors was determined using transwell inserts (BD, Saudi Arabia) with 8 *μ*m pore filters in 12-well plates as previously published [16]. Briefly, cells at a density of 5 × 10^4^ were added to the upper side of the transwell filter chamber in a 12-well plate and complete DBMSC culture medium with chemotactic factors was added to the bottom chamber. Migration observed in the presence of 30% FBS, and with medium alone, served as positive and negative controls, respectively. After overnight incubation as described above, the upper side of the filters was carefully washed with PBS. Cells remaining on the upper side of the filters were removed. Cells that had migrated to the lower side of the filter were stained with haematoxylin, and then the filter was excised from the insert and placed onto a glass slide. The total number of cells that had migrated to the lower side of the filter was counted using light microscopy at 100x magnification. Cells were blindly counted and data were expressed as numbers of total migrated cells per insert related to that of the negative control. The chemotactic activity of stromal derived factor, SDF (150 ng/mL), platelet derived growth factor, PDGF (10 ng/mL), HGF (50 ng/mL), and MCP (100 ng/mL) was determined. All chemotactic factors used were recombinant factors of human origin (R&D Systems, Saudi Arabia). The concentrations used gave an optimal migratory effect as previously published [16]. The number of replicates, cell passage, and number of placentae were as in Section 2.10.

### 2.12. Statistical Analysis

Data were analysed using the *t*-test. These analyses were performed using GraphPad Prism 5. Results were considered to be statistically significant if *P* < 0.05.

## 3. Results

### 3.1. Isolation of DBMSCs

**Figure 1:**
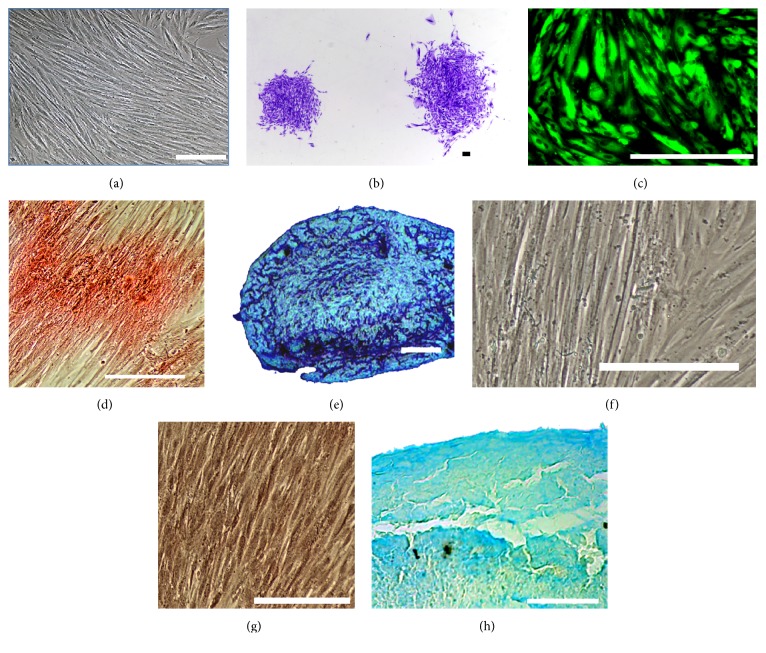
Photomicrographs showing representative examples of DBMSCs at passage three (a) and a colony forming unit of DBMSCs isolated from passage three (b). The differentiation of DBMSCs isolated from passage three into adipocytes shown by HCS LipidTOX Green neutral lipid staining of adipocytes after 21 days (c); osteocytes were shown by Alizarin Red S staining of osteocytes after 21 days (d) and chondrocytes shown by Alcian Blue staining of cross-section of a chondrogenic pellet after 21 days (e). Negative control of DMSC differentiation into adipocytes (f), osteocytes (g), and chondrocytes (h). Scale bars represent 100 *μ*m.

DBMSCs were isolated from the* decidua basalis* attached to the maternal side of human placenta and these cells formed a homogenous monolayer of adherent cells which exhibited a fibroblast-like cell morphology (Figure 1(a)). DBMSCs were prepared from placentae obtained from pregnancies with male births. Contamination by fetal derived cells in the DBMSC populations was assessed by RT-PCR detecting for the SRY gene. The RT-PCR of all DBMSCs isolated from 20 placentae did not detect the SRY gene, confirming that these DBMSC preparations consisted only of maternal-derived cells. The positive control was the detection of the SRY gene in the placental mesenchymal stem cell preparation of fetal-derived cells as previously published [16] (data not shown).

### 3.2. Colony Forming Unit Assay

Cells were clonogenic as shown by CFU assay (Figure 1(b)). Clusters of ≥50 cells were counted as colonies. Seeding cells at density of 100 cells per well resulted in 30.5 ± 5.14 of the total cells per well forming colonies, with an average of 60.5 ± 10.13 cells/colony (20 colonies counted for each sample).

### 3.3. Differentiation of DBMSCs

To determine whether DBMSCs could differentiate into multiple mesenchymal cell lineages, DBMSCs were cultured in adipogenic, osteogenic, and chondrogenic medium. The qualitative confirmation of differentiation was made by LipidTOX Green Neutral Lipid Stain for adipogenic differentiation (Figure 1(c)), Alizarin Red S staining for osteogenic differentiation (Figure 1(d)), and Alcian Blue staining for chondrogenic differentiation (Figure 1(e)).

### 3.4. Flow Cytometry Analysis of DBMSCs

**Figure 2:**
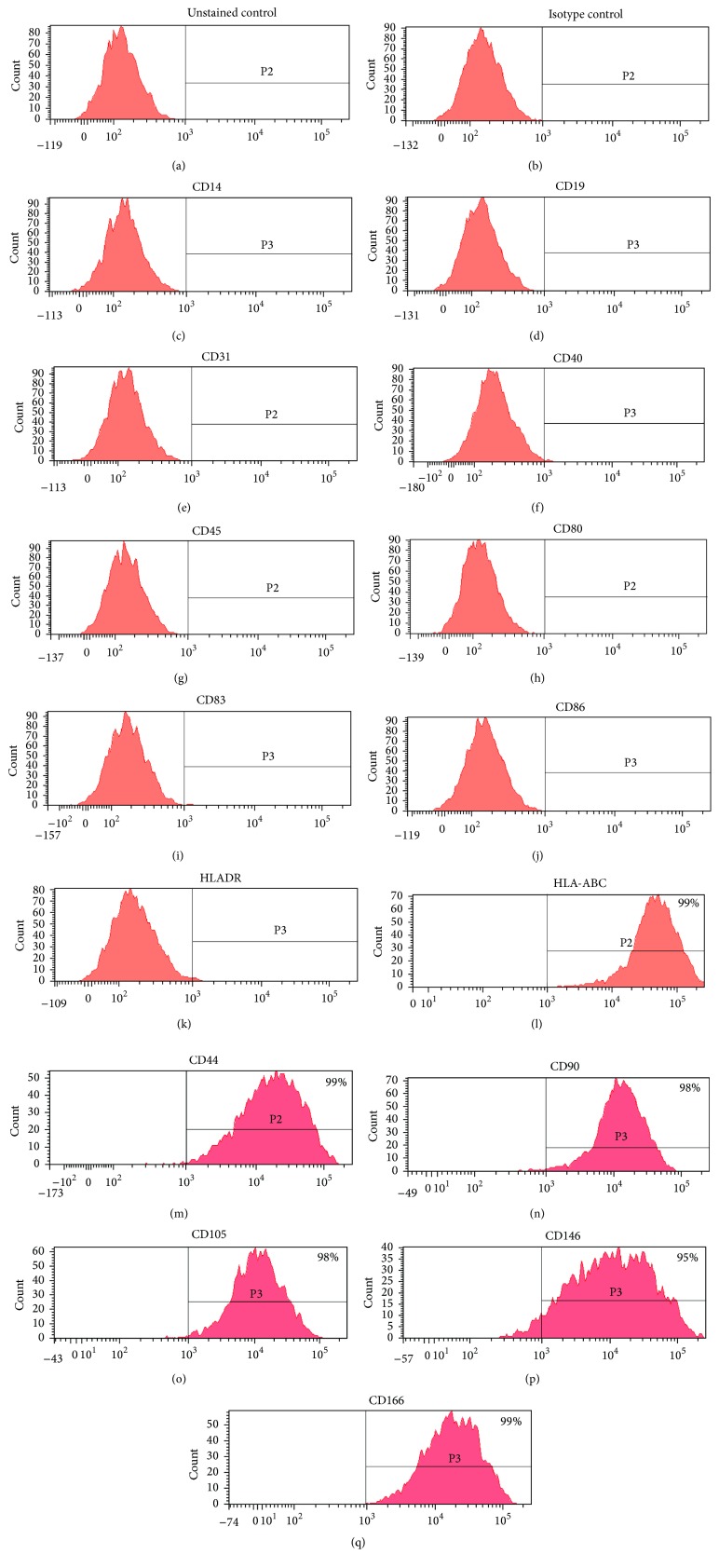
Histograms showing representative examples of DBMSCs isolated from passage three and stained with cell surface markers. DBMSCs were negative for the hematopoietic markers (CD14, CD19, and CD45), endothelial marker CD31, costimulatory molecules (CD40, CD80, CD83, and CD86), and HLA-DR. DBMSCs were positive for HLA-ABC, CD44, CD90, CD105, CD146, and CD166. Unstained and isotype controls were used ((a) and (b)). DBMSCs from passage three of 20 placentae were analyzed.

We determined the phenotype of DBMSCs by flow cytometry. Cells were positive for MSC markers (CD44, CD90, CD105, CD146, and CD166) and HLA-ABC (Figure 2). In addition, the cells were negative for the hematopoietic markers (CD14, CD19, and CD45), CD31 (endothelial marker), costimulatory molecules (CD40, CD80, CD83 and CD86), and HLA-DR (Figure 2).

### 3.5. Human DBMSCs Secrete Many Cytokines Growth Factors and Immune Proteins

MSCs secrete many cytokines and growth factors to modulate their functions (autocrine) or other cells' functions (paracrine). Consequently, we screened the conditioned medium collected from the culture of DBMSCs at passage three for the secretion of cytokines and growth factors by ELISA. We detected many cytokines and growth factors as shown in Table 1.

### 3.6. Human DBMSCs Express Many Adhesion Molecules

Human DBMSCs from passages three to five expressed a broad spectrum of adhesion molecules including integrin (*α*1, *α*2, *α*2b, *α*3, *α*4, *α*5, *α*6, *α*7, *α*8, *α*9, *α*10, *α*11, *α*D, *α*E, *α*L, *α*M, *α*V, *α*X, *β*1, *β*2, *β*3, *β*4, *β*5, *β*6, *β*7, *β*8), immunoglobulin superfamily (ICAM1, ICAM2, PECAM, VCAM, and MEDCAM), and selectin L and selectin P ligand as shown in Table 2. The level of mRNA expression did not change between passages. In addition, the flow cytometry analysis of surface protein expression by DBMSCs revealed that DBMSCs at passage three expressed ICAM-1 (CD54) (62.4%  ± 5.23%) (Table 7) and this expression changed at passages four and five (*P* < 0.05), as shown in Figure 5.

### 3.7. Human DBMSCs Express Many Chemokines/Receptors

**Figure 3:**
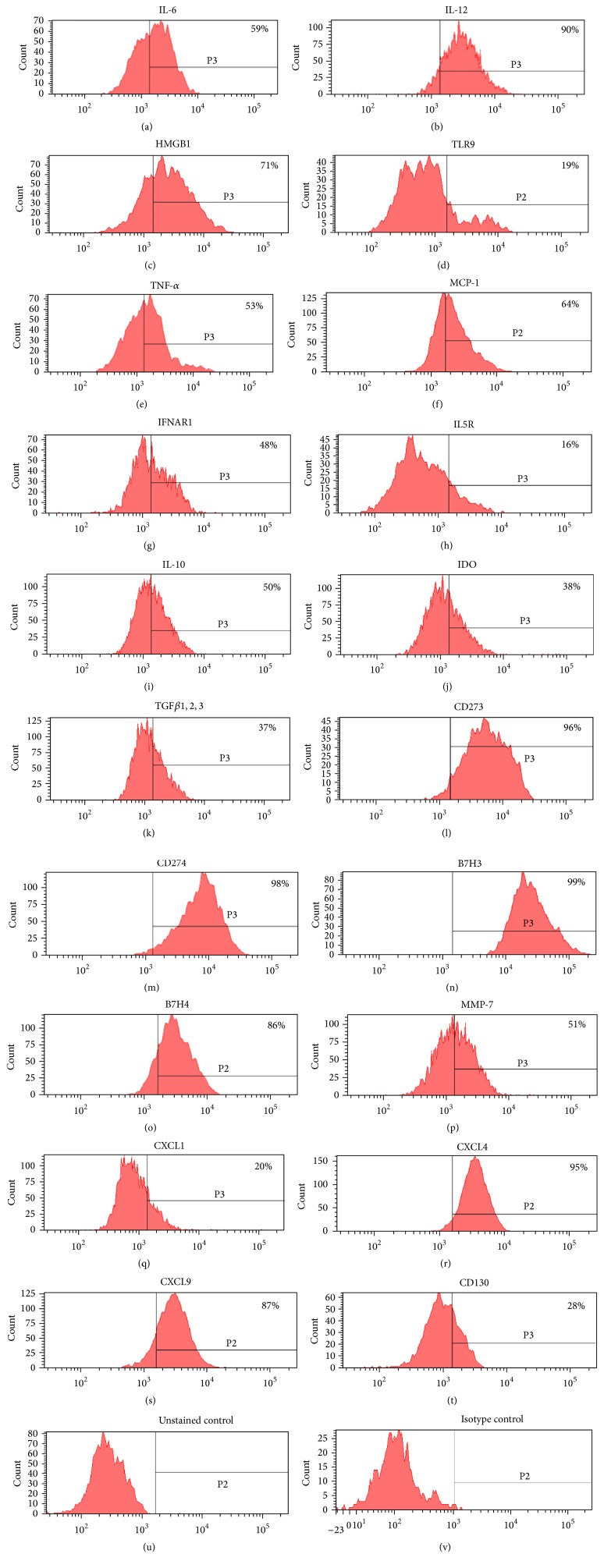
Histograms showing representative examples of DBMSCs isolated from passage three and stained with intracellular and cell surface markers. DBMSCs were positive for IL-6, IL-12, HMGB1, TLR-9, TNF-*α*, MCP-1, IFNAR1, IL5R, IL-10, IDO, TGF*β*1,2,3, CD273, CD274, B7H3, B7H4, MMP-7, CXCL1, CXCL4, CXCL9, and CD130. Unstained and isotype controls were used ((u) and (v)). DBMSCs from passage three to passage five from five individual placentae were analyzed.

Human DBMSCs from passages three to five expressed a broad spectrum of CC Chemokine/Receptor family members (CCL1, CCL2, CCL3, CCL4, CCL5, CCL11, CCL15, CCL17, CCL19, CCL21, CCL24, CCL25, CCL26, CCL27, CCR1, CCR2, CCR4, CCR5, CCR6, CCR7, and CCR10), CXC Chemokine/Receptor family (CXCL3, CXCL5, CXCL6, CXCL9, CXCL12, CXCL13, CXCL14, CXCL16, CXCR1, CXCR4, CXCR5, and CXCR6), C Chemokine/Receptor family (XCL1, XCL2, and XCR1), and CX_3_C Chemokine/Receptor family (CX3CL1 and CX3CR1) as shown in Table 4. The level of mRNA expression did not change significantly between passages. The FACS analysis showed that DBMSCs at passage three expressed some of proteins for chemokine ligands and receptors examined as shown in Table 7 and Figure 3. The expression of these proteins changed at passages four and five but was not significant (*P* > 0.05). In addition, DBMSCs lacked the expression of CCR1–CCR10, CXCR1–CXCR4, CXCR7, XCR1, and CX3CR1 (Table 7).

### 3.8. Human DBMSCs Express Many Profiles of Cytokines/Receptors

Human DBMSCs from passages three to five expressed a broad spectrum of cytokines/receptors including inflammatory IL1A, IL1*β*, IL8, IL9, IL12A, IL12B, IL13, IL15, IL16, IL17A, IL18, IL20, IL23A, IL26, IL27, IL28A, IL29, IL31, IL32, IL33, IL34, IL1R1, IL2RB, IL2RG, IL3RA, IL6R, IL7R, IL17RC, IL18R1, and IL22RA1 and anti-inflammatory cytokines/receptors IL4, IL5, IL6, IL10, IL19, IL24, IL25, IL27, IL4R, IL10RA, and IL10RB as shown in Table 5. The level of mRNA expression did not change significantly between passages. In addition, the FACS analysis showed that DBMSCs at passage three expressed proteins for some of cytokines ligands and receptors examined as shown in Table 7 and Figure 3. The expression of these proteins changed at passages four and five but was not significant (*P* > 0.05). In addition, these cells lacked the expression of IL1*β* and interleukin-7 receptor-*α* (CD127).

### 3.9. Human DBMSCs Express Many Growth Factor Receptors and Proteolytic Enzyme of the Extracellular Matrix

Human DBMSCs from passages three to five expressed a broad spectrum of growth factor receptors including platelet-derived growth factor receptors *α* and *β* (PDGFR*α* and *β*), fibroblast growth factor receptors 2 and 4 (FGFR2 and 4), interferon gamma receptors 1 and 2 (IFNGR1 and 2), interferon alpha, beta, and omega receptors 1 and 2 (IFNAR1 and 2), nerve growth factor receptor (NGFR), kinase insert domain receptor (type III receptor tyrosine kinase) or KDR, interferon, kappa (IFNK), fibroblast growth factor receptors 1 and 3 (FGFR1 and 3), transforming growth factor beta receptors 1 and 2 (TGFBR1 and 2), formyl peptide receptor 2 (FPR2), opioid growth factor receptor (OGFR), insulin-like growth factor 1 receptor (IGF1R), colony stimulating factor 2 receptor beta (CSF2RB), epidermal growth factor receptor (EGFR), neuroplastin (NPTN), histamine receptor H4 (HRH4), fms-related tyrosine kinase 4 (FLT4), fms-related tyrosine kinase 1 (vascular endothelial growth factor or FLT1), tumor necrosis factor receptor superfamily member 1A (TNFRSF1A), tumor necrosis factor receptor superfamily member 1B (TNFRSF1B), and met protooncogene (hepatocyte growth factor receptor or MET) as shown in Table 6. The level of mRNA expression did not change significantly between passages. In addition, the FACS analysis showed that DBMSCs at passage three expressed proteins for some of growth factor receptors examined as shown in Table 7 and Figure 3. The expression of these proteins changed at passages four and five but was not significant (*P* > 0.05). However, the expression of IFN-*γ* and TGF-*β*1 by DBMSCs significantly changed from passages 3–5 (*P* < 0.05) as shown in Figure 5. In addition, these cells lacked the expression of GMCSF receptor or CD116.

### 3.10. Human DBMSCs Express Immune Suppressive Genes, Negative Cosignalling Proteins, and Other Immune Proteins

DBMSCs from passages three to five expressed the immunosuppressive enzyme indoleamine-2,3-dioxygenase (IDO). The level of mRNA expression did not change significantly between passages. In addition, the flow cytometry analysis revealed that DBMSCs at passage three expressed proteins for some of immune factors examined as shown in Table 6 and Figure 3. The expression of these proteins changed at passages four and five but was not significant (*P* > 0.05).

### 3.11. The Proliferation Potential of DBMSCs

**Figure 4:**
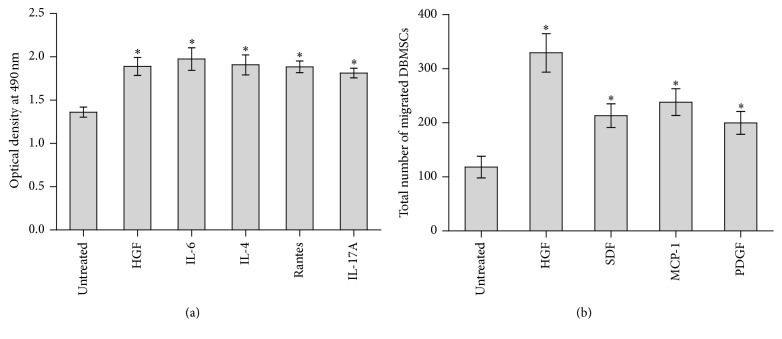
The effect of different cytokines on the proliferation of DBMSCs (a) and the effect of different chemotactic factors (HGF, SDF, MCP-1, and PDGF) on the migration of DBMSCs (b). The proliferation of DBMSCs was significantly increased in the presence of HGF, IL-6, IL-4, Rantes, and IL-17A (^*∗*^ *P* < 0.05). The chemotactic factors significantly (^*∗*^ *P* < 0.05) stimulated the migration of DBMSCs in transwell migration plates. DBMSCs at passage three from five individual placentae were analyzed. Bars represent standard errors.

**Figure 5:**
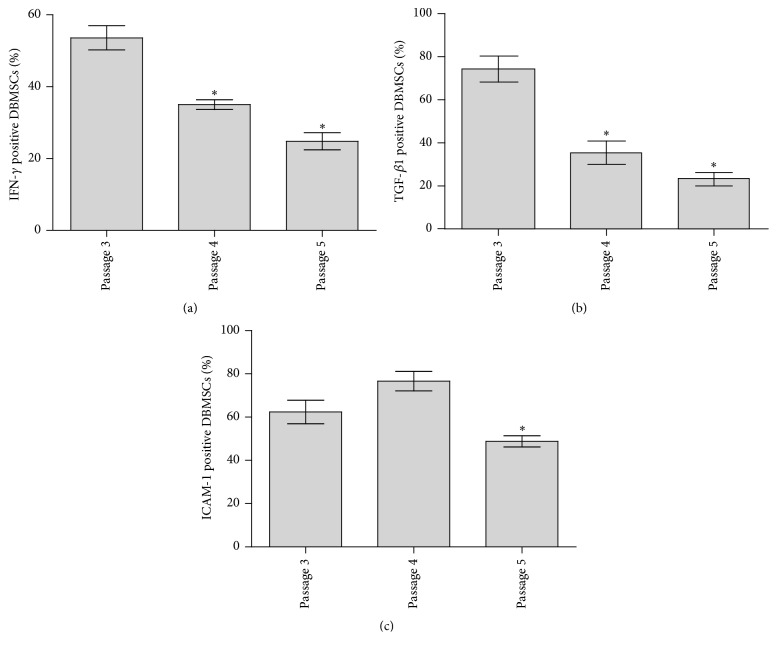
Flow cytometric analysis of the expression of interferon gamma (IFN-*γ*), transforming growth factor-*β* (TGF-*β*), and intercellular adhesion molecule-1 (ICAM-1) by human* decidua basalis* mesenchymal stem cells (DBMSCs). The expression profiles of five individual experiments demonstrating that the expression of IFN-*γ*, TGF-*β*1, and ICAM-1 by DBMSCs significantly changed from passage 3 to passage 5. The levels of expression are presented as mean percentage of positive cells as determined by flow cytometry. Experiments were conducted in triplicate using the indicated DBMSC passages. Five independent placentae were used to prepare DBMSCs. ^*∗*^ *P* < 0.05. Bars represent standard errors.

To investigate whether DBMSCs can proliferate in response to a number of proliferative stimuli, an MTS assay was used. The results revealed that HGF, IL-6, IL-4, Rantes, and IL-17A significantly increased (*P* < 0.05) the proliferation of DBMSCs compared with the untreated control, as shown in Figure 4.

### 3.12. The Migration Potential of DBMSCs

The migration potential of DBMSCs, in response to a number of chemotactic factors, was evaluated using a transwell migration assay. The results revealed that these chemotactic factors stimulated the migration of DBMSCs. The number of migrated DBMSCs increased significantly (*P* < 0.05) in the presence of HGF (329.3 ± 35.49), SDF (213.2 ± 21.91), MCP-1 (238.2 ± 24.80), and PDGF (199.8 ± 20.87), compared with the untreated DBMSCs control (118.2 ± 35.49) (Figure 4).

## 4. Discussion

### 4.1. Isolation of Mesenchymal Stem Cells from the* Decidua Basalis* of Human Term Placenta

In this study, we isolated MSCs from the* decidua basalis* tissue attached to the maternal side of human placenta. We cultured DBMSCs for five passages. Morphologically, these cells formed a homogenous monolayer of adherent cells that exhibited fibroblast-like cell morphology from passage two onward (Figure 1(a)). In addition, these DBMSCs were positive for HLA-ABC and MSC markers and were negative for the hematopoietic markers, the endothelial cell marker, costimulatory molecules, and HLA-DR (Figure 2). The expression of these molecules by DBMSCs did not change throughout culture. In addition, these DBMSCs exhibited multipotent differentiation and clonogenic ability (Figure 1). These data confirm that these cells have stem cell-like properties and meet the minimal criteria for defining multipotent MSC [11].

### 4.2. DBMSCs Express a Specific Combination of Adhesion Molecules

Cell surface adhesion molecules play essential roles in several cellular processes, such as cell growth, differentiation, migration, and immune responses. In addition, adhesion molecules are classified into four major families including the immunoglobulin (Ig) superfamily cell adhesion molecules (CAMs), integrins, cadherins, and selectins [30]. In this study, we studied the expression of adhesion molecules by DBMSCs. We have shown for the first time the expression of a broad range of adhesion molecules by DBMSCs (Table 2). However, the expression of ICAM-1 by DBMSCs was previously reported [31, 32]. In addition, DBMSCs share the expression of these adhesion molecules with pMSCs and BMMSCs [16, 33], but they also express additional adhesion molecules including *α*9, *α*D, *α*L, *α*X, *β*2, and MEDCAM (Table 3). The role of these molecules in DBMSC functions remains to be assessed in a future study. However, these adhesion molecules may contribute to the structural and functional maintenance of DBMSCs in their microenvironment (niche) [34] or regulate the division and differentiation of DBMSCs or mediate DBMSC immune functions on immune cells as it has been demonstrated for *α*5*β*1-integrin, ICAM-1, and VCAM-1 [35, 36].

### 4.3. DBMSCs Express a Specific Combination of Chemokines/Receptors

MSCs have a great therapeutic potential in treating human diseases. During the course of stem cell transplantation, transplanted MSCs home to the site of injury in a process known as homing, which involves cell recruitment, migration, and adhesion [37]. Chemotaxis plays an essential role in homing of circulating MSCs to peripheral tissues. Chemotactic process depends on the signaling protein molecules called chemokines [37]. Chemokines are a family of structurally related proteins that is divided into four subfamilies: CXC, CC, C, and CX_3_C chemokines. Chemokine receptors are classified as G protein coupled receptors for CXC, CC, C, or CX_3_C chemokines [38]. Chemokines participate in many biological processes such as the regulation of inflammation, differentiation of cells, the migration of cells, and angiogenesis, the formation of new blood vessels [39]. In response to proinflammatory stimuli, the expression and secretion of chemokines are induced in cells in order to attract and activate immune cells such as neutrophils, monocytes/macrophages, and lymphocytes to the site of injury [40]. We and others have shown that MSCs express and secrete many chemokines [16, 41].

The expression profile of chemokines by DBMSCs is largely unknown. In this study, we studied the expression of chemokines by DBMSCs. We showed that DBMSCs express many chemokines which belong to CC, CXC, C, and CX_3_C families (Tables 4 and 7 and Figure 3). Others have shown that DBMSCs express and secrete MCP-1, CXCL-1, CXCL-8, CXCL-12, and CXCL13 [31, 32, 42]. DBMSCs and pMSCs share the expression of most of the chemokine ligands listed in Table 4 except that DBMSCs express additional ligands including CCL11, CCL15, CCL17, CCL19, CXCL9, and CXCL13 (Table 4) while they lack the expression of others including CCL7, CCL8, CCL18, CXCL10, and CXCL11 [16]. In addition, DBMSCs, pMSCs, and BMMSCs share the expression of many chemokine receptors including CCR1, CCR2, CCR4, CCR6, CCR7, CXCR1, CXCR4, CXCR5, CXCR6, and CX3CR1 but DBMSCs also express additional receptors including CCR10 and XCR1 (Table 4) and lack the expression of CCR3 and CXCR3 [16, 33, 43].

The significance of chemokine expression by DBMSCs needs to be further studied. However, it may account for their migration ability as we showed in this study that DBMSCs can migrate in response to HGF, SDF, MCP-1, and PDGF* in vitro*. The distinct chemokine expression profile of DBMSCs may also account for their ability to modulate the functions of immune cells since they express many chemokines that are known for their immune modulatory effects such as MCP-1 which can modulate the immune response by inducing apoptosis in T cells, switching the differentiation of T cells from Th0 to Th2, generating regulatory T cells, inhibiting the production of antibodies by plasma cells, and inhibiting the function of the proinflammatory Th17 cells [44]. In addition, DBMSCs express chemokines with activity to induce angiogenesis such as MCP-1 and CXCL12-CXCR4 receptor complex [44–48]. Interestingly, DBMSCs also express CXCL9 which is known for its function against angiogenesis and liver fibrosis [49]. Therefore, DBMSCs express molecules with contrasting functions suggesting that these cells may have multiple functions as suggested by others that MSCs can be immunosuppressive or immunostimulatory and this functional switch of MSCs depends on the microenvironment [50]. Here, we discussed examples of few chemokines expressed by DBMSCs demonstrating their possible roles in DBMSC functions. Therefore, DBMSCs could potentially have beneficial therapeutical effects in the treatment of many of human diseases such as cancer, cardiovascular, immunological, and liver diseases. However, future functional studies are necessary to determine the exact roles of these chemokines in DBMSC functions.

### 4.4. DBMSCs Express a Specific Combination of Cytokines, Growth Factors, and Immune Molecules

Cytokines are membrane bound or secreted proteins released by many cells. Cytokines can modulate the functions of many cells, such as immune modulation, migration, proliferation, and differentiation. Our knowledge regarding the expression profile of cytokines produced by DBMSCs is largely unknown. Therefore, this study was performed to demonstrate that DBMSCs express and secrete a distinct profile of cytokines (Tables 1 and 5). pMSCs and BMMSCs express and also secrete most of these cytokines [16, 51, 52]. The functional consequences of DBMSC expression or secretion of cytokines are unknown. DBMSCs may use these cytokines to mediate their immunosuppressive functions as it has been recently reported that DBMSCs ameliorate T helper 1 cell induced preeclampsia-like symptoms in mice via the secretion of IL-6, VEGF, and TGF-*β* [53]. However, MSCs also have an immunostimulatory property, which in the case of BMMSCs is not constitutively present, but this immunomodulatory property is detected following stimulation by inflammatory mediators secreted by immune cells and inflamed tissue [54]. BMMSCs are polarized toward either a proinflammatory (MSC-1) phenotype or an anti-inflammatory (MSC-2) phenotype via a mechanism that is mediated by the activation of TLRs [55]. In this study, we demonstrated that DBMSCs express and secrete a unique profile of proinflammatory and anti-inflammatory cytokines. In addition, they express TLRs. Therefore, these cells may function as immunostimulatory or immunosuppressive cells depending on the microenvironment. Consequently, it is important to determine the factors that trigger immunostimulation or immunosuppression in DBMSCs since the required stem cell immune status may differ depending on the intended therapeutic application.

In this study, we have also demonstrated a profile of expression/secretion of many growth factors/receptors, coinhibitory molecules (CD273, CD274, B7H3, and B7H4), and the immune suppressive enzyme IDO (Tables 6 and 7). This profile shares similarities and differences with the profile we previously reported for pMSCs [16]. The significance of this expression/secretion profile should be addressed in future functional studies. These molecules have modulated cellular functions including angiogenesis, proliferation, migration, adhesion, apoptosis, antitumor activity, antifibrotic activity, and immune modulatory activities. One example of how DBMSCs use these molecules to modulate functions comes from a recent study when IDO was shown to modulate the suppressive effect of DBMSCs on T cell functions in a mouse model of preeclampsia [53].

## 5. Conclusion

This is the first study to show that DBMSCs express an extensive range of molecules that are implicated in many cellular functions. The diverse expression of molecules by DBMSCs makes them a worthwhile candidate for future functional studies to determine their therapeutic potential in treating human diseases such as cancers, immune disorders, and liver and cardiovascular diseases.

## Figures and Tables

**Table 1:** Soluble factor secreted by DBMSCs as determined by sandwich ELISA. Each experiment was performed using DBMSCs from passages three of five individual placentae.

Soluble factors	Concentration
IL1*β*	10.38 ± 1.46
IL1ra	76.25 ± 5.54
IL-3	0^a^
IL-5	0
IL-6	575.0 ± 32.27
sIL-6R	166.30 ± 28.90
IL-10	9.45 ± 0.68
IL12p70	10.98 ± 1.48
IL-19	38 ± 2.85
VEGF	755 ± 21.02
sVEGFR	0
IFN-*γ*	18 ± 0.91
MCSF	853.80 ± 112
GMCSF	26.50 ± 6.43
MIF	2.85 ± 0.15^a^
HGF	148.8 ± 17.37
TGF*β*-1	895 ± 29.30
LIF	0
B7H4	11.67 ± 1.28^a^
IDO	0^a^
EGF	0

*Note*. (a) IL-3, MIF, B7H4, and IDO were expressed in ng/mL whereas all other concentrations are in pg/mL.

**Table 2:** The expression of adhesion molecules in DBMSCs as determined by real-time PCR. Each experiment was performed using DBMSCs from passages three of five individual placentae.

Adhesion molecules	Result	Adhesion molecules	Results	Adhesion molecules	Results
Integrin, alpha 1	++	Integrin, alpha L (CD11A)	+++	PECAM (CD31)	+
Integrin, alpha 2 (CD49B)	++	Integrin, alpha M (CD11b)	++	VCAM (CD106)	+
Integrin, alpha 2b (CD41)	+	Integrin, alpha V (CD51)	+++	MEDCAM	+++
Integrin, alpha 3 (CD49C)	+++	Integrin, alpha X (CD11c)	++	SELE: selectin E	−
Integrin, alpha 4 (CD49D)	++	Integrin, beta 1 (CD29)	+++	SELL: selectin L	+
Integrin, alpha 5 (CD49e)	+++	Integrin, beta 2 (CD18)	++	SELP: selectin P (CD62)	−
Integrin, alpha 6 (CD49f)	+++	Integrin, beta 3 (CD61)	+++	SELPLG: selectin P ligand	+++
Integrin, alpha 7	+++	Integrin, beta 4 (CD104)	+++	SELD: selectin D	−
Integrin, alpha 8	+++	Integrin, beta 5	+++		
Integrin, alpha 9	+++	Integrin, beta 6	+++		
Integrin, alpha 10	+++	Integrin, beta 7	+++		
Integrin, alpha 11	+++	Integrin, beta 8	+++		
Integrin, alpha D	+	ICAM1 (CD54)	+++		
Integrin, alpha E (CD103)	++	ICAM2 (CD102)	+++		

*Note*. (−) no expression; (+) weak expression; (++) moderate expression; (+++) strong expression of mRNA.

**Table 3:** The unique expression of adhesion molecules and chemokines in DBMSCs as determined by real-time PCR. Each experiment was performed using DBMSCs from passages three of five individual placentae.

Adhesion molecules	Result	Chemokines and chemokine receptors	Results
Integrin, alpha 9	+++	CCL11	+
Integrin, alpha D	+	CCL15	+
Integrin, alpha L (CD11A)	+++	CCL17	+
Integrin, alpha X (CD11c)	++	CCL19	+++
Integrin, beta 2 (CD18)	++	CXCL9	++
MEDCAM	+++	CXCL13	+
		CCR10	+++
		XCR1	+

**Table 4:** The expression of chemokine receptors and chemokine ligands in DBMSCs as determined by real-time PCR. Each experiment was performed using DBMSCs from passages three of five individual placentae.

Chemokines	Results	Chemokines	Results	Chemokines	Results
CCL1	++	CCL19	+++	CXCL9	++
CCL2	++	CCL20	−	CXCL10	−
CCL3	++	CCL21	+++	CXCL11	−
CCL4	+++	CCL22	−	CXCL12	+++
CCL5	++	CCL23	−	CXCL13	+
CCL7	−	CCL24	++	CXCL14	+++
CCL8	−	CCL25	+++	CXCL16	+++
CCL11	+	CCL26	+++	XCL1	++
CCL13	−	CCL27	+	XCL2	+++
CCL14	−	CCL28	−		
CCL15	+	CX3CL1	+++		
CCL16	−	CXCL3	+		
CCL17	+	CXCL5	++		
CCL18	−	CXCL6	+		

Chemokine receptors	Results	Chemokine receptors	Results	Chemokine receptors	Results

CCR1	++	CCR7	+++	CXCR3	−
CCR2	+	CCR8	−	CXCR4	+
CCR3	−	CCR9	−	CXCR5	+
CCR4	+	CCR10	+++	CXCR6	+++
CCR5	++	CXCR1	++	CX3CR1	+++
CCR6	+++	CXCR2	−	XCR1	+

*Note*. (−) no expression; (+) weak expression; (++) moderate expression; (+++) strong expression of mRNA.

**Table 5:** The expression of cytokine mRNA in DBMSCs as determined by real-time PCR. Each experiment was performed using DBMSCs from passages three of five individual placentae.

Cytokines	Results	Cytokines	Results	Cytokines	Results
IL1A	+++	IL13	+++	IL26	−
IL1B	+++	IL15	+++	IL27	+++
IL2	−	IL16	+++	IL28A	+
IL3	−	IL17A	+	IL29	++
IL4	++	IL18	+++	IL31	++
IL5	++	IL19	+++	IL32	+++
IL6	+++	IL20	+++	IL33	+++
IL8	+++	IL21	−	IL34	+++
IL9	+	IL22	−		
IL10	++	IL23A	+++		
IL12A	+++	IL24	+++		
IL12B	++	IL25	++		

Cytokine receptors	Results	Cytokine receptors	Results	Cytokine receptors	Results

IL1R1	+++	IL6R	+++	IL18R1	+++
IL2RA	−	IL7R	++	IL22RA1	+
IL2RB	+++	IL9R	−	IL22RA2	−
IL2RG	+	IL10RA	++	IL18R1	−
IL3RA	++	IL10RB	+++		
IL4R	+++	IL17RC	++		

*Note*. (−) no expression; (+) weak expression; (++) moderate expression; (+++) strong expression of mRNA.

**Table 6:** The expression of growth factors and growth factor receptors in DBMSCs as determined by real-time PCR. Each experiment was performed using DBMSCs from passages three of five individual placentae.

Growth factor receptors	Results	Growth factor/receptors and immune molecule	Results
PDGFRA: platelet-derived growth factor receptor *α*	+++	FPR2: formyl peptide receptor 2	++
PDGFRB: platelet-derived growth factor receptor *β*	+++	OGFR: opioid growth factor receptor (OGFR)	+++
FGFR4: fibroblast growth factor receptor 2	+++	IGF1R: insulin-like growth factor 1	+++
FGFR2: fibroblast growth factor receptor 4	++	CSF2RB: Colony stimulating factor 2 receptor, *β*	+++
IFNGR1: interferon gamma receptor 1	+++	EGFR: epidermal growth factor receptor	+++
IFNGR2: interferon gamma receptor 2	+++	NPTN: neuroplastin	+++
IFNAR1: interferon (alpha, beta, and omega) receptor 1	+++	HRH4: histamine receptor H4	+
IFNAR2: interferon (alpha, beta, and omega) receptor 2	+++	FLT4: fms-related tyrosine kinase 4	+++
NGFR: nerve growth factor receptor	+++	FLT1: fms-related tyrosine kinase 1 (vascular endothelial growth factor)	+++
KDR: kinase insert domain receptor (type III receptor tyrosine kinase)	++	TNFRSF1A: tumor necrosis factor receptor superfamily member 1A	+++
IFNK: interferon, kappa	+	TNFRSF1B: tumor necrosis factor receptor superfamily member 1B	+++
FGFR1: fibroblast growth factor receptor 1	+++	MET: met protooncogene (hepatocyte growth factor receptor)	+++
FGFR3: fibroblast growth factor receptor 3	+	EGF: epidermal growth factor	−
TGFBR1: transforming growth factor beta receptor 1	+++	Indoleamine 2,3-dioxygenase	+
TGFBR2: transforming growth factor beta receptor II	+++		

*Note*. (−) no expression; (+) weak expression; (++) moderate expression; (+++) strong expression of mRNA.

**Table 7:** The mean expression of the percentage of immune proteins in DBMSCs as determined by flow cytometry.

Markers	Passage 3	Passage 4	Passage 5	Markers	Passage 3	Passage 4	Passage 5
IL-1*β*	—	—	—	D6	10.13 ± 2.52	13.03 ± 6.28	12.30 ± 4.02
IL-6	59.70 ± 1.03	63.00 ± 0.77	64.60 ± 5.40	CCR1	—	—	—
IL-8 (CXCL8)	14.4 ± 2.51	10.4 ± 0.51	17.4 ± 1.51	CCR2	—	—	—
IL-10	50.77 ± 4.08	43.43 ± 8.15	45.73 ± 2.46	CCR3	—	—	—
IL-12	90.00 ± 6.90	87.95 ± 7.05	71.35 ± 9.25	CCR4	—	—	—
TNF-*α*	53.37 ± 2.7	53.97 ± 10.95	54.00 ± 4.23	CCR5	—	—	—
MCP-1	64.47 ± 5.98	77.40 ± 7.61	81.30 ± 8.20	CCR6	—	—	—
HMGB-1	71.33 ± 3.69	72.63 ± 12.39	65.5 ± 8.72	CCR7	—	—	—
IFN-*γ*	53.60 ± 3.24	35.10 ± 1.170	24.4 ± 2.884	CCR8	—	—	—
IFNAR1	48.87 ± 2.63	51.50 ± 3.72	41.47 ± 4.26	CCR9	—	—	—
IFNAR2	73.43 ± 18.09	94.10 ± 1.89	93.13 ± 2.72	CCR10	—	—	—
TLR3	13.13 ± 5.79	13.47 ± 3.24	17.27 ± 2.83	CXCR1	—	—	—
TLR7	8.76 ± 3.06	10.43 ± 6.22	8.73 ± 2.92	CXCR2	—	—	—
TLR9	19.80 ± 3.18	16.37 ± 5.03	23.13 ± 3.05	CXCR3	—		
IL5-R	16.50 ± 1.041	9.86 ± 5.13	10.43 ± 3.53	CXCR4	—	—	—
IL-7R (CD127)	—	—	—	CXCR7	—	—	—
TGF B1	74.40 ± 6.01	35.60 ± 5.50	23.30 ± 2.81	XCR1	—	—	—
TGFB1,2,3	37.00 ± 1.07	30.03 ± 5.76	37.40 ± 5.09	CX3CR1	—	—	—
MMP7	51.70 ± 3.85	63.73 ± 13.45	57.10 ± 2.81	CXCL1	20.07 ± 2.73	19.07 ± 5.05	25.53 ± 6.98
ICAM-1 (CD54)	62.4 ± 5.234	76.6 ± 4.022	48 ± 2.609	CXCL4	95.50 ± 1.73	97.07 ± 0.94	95.67 ± 3.08
CD116	—	—	—	CXCL5	7.86 ± 3.32	5.63 ± 1.75	5.00 ± 1.12
CD118	14.83 ± 4.95	17.03 ± 9.81	12.87 ± 4.36	CXCL9	87.67 ± 1.13	87.70 ± 8.32	92.67 ± 5.55
CD120a	11.27 ± 3.78	12.97 ± 8.24	4.60 ± 2.01	IP10 (CXCL10)	9.40 ± 2.45	4.03 ± 1.36	3.96 ± 1.15
CD120b	10.57 ± 4.23	27.53 ± 20.49	5.36 ± 0.39	CXCL12	15.83 ± 2.00	13.40 ± 3.80	21.33 ± 7.45
CD130	28.30 ± 4.56	50.63 ± 22.36	38.33 ± 12.29	CX3CL1	10.30 ± 4.18	8.13 ± 2.31	6.23 ± 0.44
CD134	18.43 ± 5.49	15.33 ± 6.25	11.20 ± 1.05	CCL5 (RANTES)	13.93 ± 5.38	5.93 ± 2.22	6.13 ± 4.04
CD234	9.96 ± 0.77	8.93 ± 4.65	7.33 ± 2.31	CCL11	9.90 ± 4.25	8.23 ± 4.48	6.60 ± 1.55
CD273	96.83 ± 0.66	90.73 ± 6.39	86.50 ± 9.95				
B7H1 (CD274)	98.00 ± 0.21	91.80 ± 3.63	85.60 ± 2.01				
CD276 (B7H3)	99.97 ± 0.03	99.93 ± 0.06	99.87 ± 0.088				
B7H4	86.43 ± 1.18	72.30 ± 4.29	82.10 ± 6.97				
IDO	38.37 ± 3.73	31.50 ± 5.90	36.97 ± 1.37				

*Note*. CD130: IL-6 Receptor Associated Transducer; CD116: GMCSF receptor; CD118: Leukemia Inhibitory Factor Receptor; MMP7: Matrix Metalloproteinase 7; D6: Chemokine Decoy Receptor.
